# Brucellosis-Related Hepatic Abscess Case

**DOI:** 10.1590/0037-8682-0093-2022

**Published:** 2022-11-21

**Authors:** Sevil Alkan, Emine Kübra Dindar Demiray

**Affiliations:** 1 Canakkale Onsekiz Mart University, Faculty of Medicine, Department of Infectious Disease, Canakkale, Turkey.; 2 Bitlis State Hospital, Department of Infection Disease and Clinical Microbiology, Bitlis, Turkey.

**FIGURE 1:**
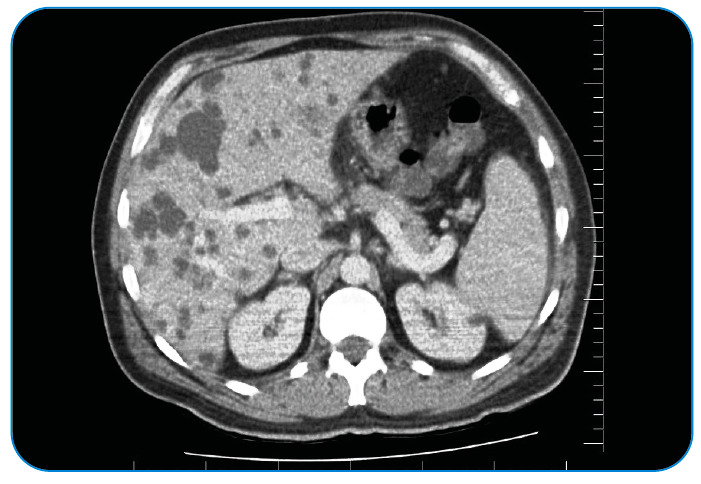
Multiple hepatic abscess in the abdominal CT scan.

**FIGURE 2:**
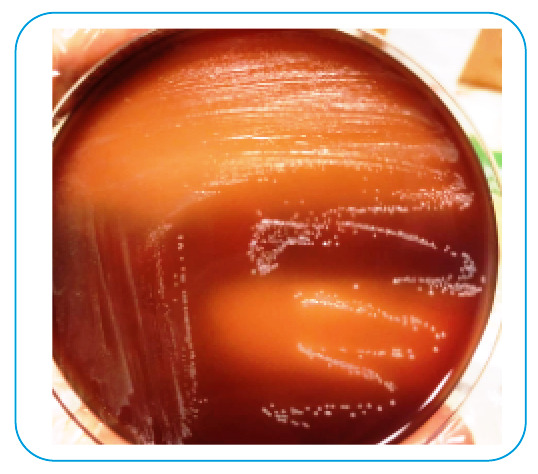
Brucella growth on the chocolate agar plate.

A 45-year-old man complained of persistent right upper abdominal pain, chills, and fever for 15 days. He had a medical history of diabetes mellitus and arterial hypertension but no record of animal contact, consumption of unpasteurized milk, or trauma. Abdominal examination revealed tenderness, hepatomegaly, and splenomegaly in the right upper abdomen. The white blood cell count was 7,900/mm^3^ (neutrophils 52% and lymphocytes 43%), hemoglobin 11 g/ mm^3^, platelets 162,109/mm^3^, and C-reactive protein 98 mg/L (0-5 mg/L). The Brucella tube agglutination test result was positive at 1:1,280. Multiple hepatic abscesses were observed on abdominal computed tomography (CT) (Figure 1). A peripherally located abscess was drained under ultrasound guidance from the radiology department. *Brucella spp.* were detected in two sets of blood samples (BACTEC Plus Aerobic/F and Anaerobic/F) and abscess drainage cultures after >48 h of incubation (Figure 2). He was diagnosed with brucellosis based on laboratory, clinical, and imaging findings. The patient received doxycycline (2×200 mg), rifampicin (1×600 mg), and streptomycin (1 g/intramuscular) during the first 2 weeks of therapy. The symptoms decreased after 5 days of treatment. In the first month of treatment, the titer decreased to 1:1,160, and the control CT also improved.

Brucellosis is a zoonotic disease that affects many organs globally, especially in developing countries. Although rare, brucellosis can cause unique or multiple^3^ hepatic abscesses (hepatic brucelloma)^1^ ^,^ ^2^. The disease can be resolved without surgical treatment^2^ ^,^ ^3^. Nevertheless, this rare manifestation of brucellosis should be considered during the differential diagnosis of hepatic abscesses.
